# Clinical, magnetic resonance imaging, and histological description of a choroid plexus papilloma with disseminated intraventricular and spinal cerebrospinal fluid drop metastases in a young adult dog: a case report

**DOI:** 10.3389/fvets.2023.1223729

**Published:** 2023-08-03

**Authors:** Guillaume Marc Albertini, Alexandra Malbon, Anne Staudacher, Fabio Stabile

**Affiliations:** ^1^Department of Neurology and Neurosurgery, Southfields Veterinary Specialists, Linnaeus Veterinary Limited, Cranes Point, Basildon, United Kingdom; ^2^Easter Bush Pathology, The Royal (Dick) School of Veterinary Studies and the Roslin Institute, Edinburgh, United Kingdom; ^3^Department of Diagnostic Imaging, Southfields Veterinary Specialists, Linnaeus Veterinary Limited, Cranes Point, Basildon, United Kingdom

**Keywords:** choroid plexus tumor, choroid plexus papilloma, drop metastases, CSF, dogs

## Abstract

A 2-year-old male entire Cane Corso was presented for investigations into a 1-week history of ambulatory paraparesis and pelvic limb ataxia gradually deteriorating. Magnetic resonance imaging (MRI) revealed intraventricular space-occupying lesions affecting the fourth ventricle and lateral apertures and intradural-extramedullary space-occupying lesions at the level of C7 vertebra, L4-L5, and L7-S1 intervertebral disk spaces. Due to poor quality of life, the patient was euthanized. A post-mortem examination revealed partially encapsulated, multifocally infiltrative, and moderately cellular neoplastic masses. The histological description was similar for all masses. The cells appeared cuboidal with round central nuclei and a moderate amount of eosinophilic cytoplasm and were arranged almost exclusively in single-layered papilliform patterns supported by a fibrovascular stroma. Mitoses were rarely observed (1/2.37 mm^2^). The primary neoplasm was morphologically most consistent with a choroid plexus papilloma despite drop metastases. This is the first report of a histologically confirmed primary ventricular choroid plexus papilloma causing disseminated MRI-apparent intraventricular and spinal drop metastases.

## Introduction

The choroid plexi are formed by modified ependymal cells and vascular proliferation of pia matter vessels (1). These cells are involved in the passive and active secretion of the cerebrospinal fluid (CSF) into the ventricular system (1). The choroid plexi are in the lateral, third, and fourth ventricles (1). Choroid plexus tumors (CPT) account for approximately 7–10% of the central nervous system (CNS) neoplasia in dogs and affect the fourth ventricle in 46% of CPTs (2–4). Due to their histological similarity to their human counterpart, CPTs are usually graded according to the World Health Organization (WHO) classification of CNS neoplasia (3, 5–7). The WHO current classification recognizes three grades of CPTs: choroid plexus papilloma (CPP, grade I), atypical CPP (aCPP, grade II), and choroid plexus carcinoma (CPC, grade III) (3, 5–7). Differentiation between CPP and aCPP is based on histological features such as increased mitotic figures, increased cellularity, nuclear atypia, and loss of papillary pattern (5–9).

In a clinical setting, differentiation between CPP and CPC involves CSF analysis and magnetic resonance imaging (MRI) features (2, 4, 5, 8, 10). A CSF total protein concentration above 80 mg/L and MRI presence of CSF drop metastases are strongly suggestive of CPC (4).

CSF drop metastases refer to the metastatic spreading of a primary CNS neoplasia to the subarachnoid space and ventricular system of the CNS, following the CSF flow (4, 11, 12). In human medicine, CSF drop metastases secondary to CPP have been reported, though rarely (13, 14). In the veterinary literature, two case reports of spinal CPP without the identification of a primary CPP have been published (15, 16). There are only two reports in the veterinary literature of a single small subarachnoid metastasis secondary to CPP only observed on post-mortem examination (4, 7). In both reports, no MRI-apparent metastasis indicating the presence of CSF drop metastasis secondary to CPP could be observed (4, 7). There is currently no evidence in the veterinary literature of histologically confirmed CPP causing disseminated MRI-apparent intraventricular and spinal CSF drop metastases.

This case report is the first clinical, MRI, and histological description of a primary ventricular canine CPP and disseminated CSF drop metastases to the ventricular (CPP) and spinal (aCPP) subarachnoid space in a young adult dog.

## Case description

A 2-year-old male entire Cane Corso was presented for investigations into a 1-week history of progressive ambulatory paraparesis and general proprioceptive ataxia in the pelvic limbs associated with spinal pain. No significant medical history was reported.

On presentation, the general examination was unremarkable.

The neurological examination revealed a normal mental status, wide-based stance in the pelvic limbs, marked ambulatory paraparesis, and general proprioceptive ataxia in the pelvic limbs. Postural reactions (paw placement and hopping) were delayed in the pelvic limbs. Spinal reflexes were normal in the pelvic limbs. The postural reactions and spinal reflexes in the thoracic limbs could not be assessed reliably due to the patient's temperament. The cranial nerve assessment was unremarkable. The discomfort was noticed upon palpation of the head and vertebral column multifocally. The neuroanatomical localization was to the T3-L3 spinal cord segments.

## Investigations

Consent was obtained to perform further investigations with magnetic resonance imaging under general anesthesia. The patient was premedicated using 0.2 mg/kg IV methadone (Comfortan^®^, Dechra) and 5 μg/kg IV medetomidine (Sedator^®^, Dechra). Induction was performed using propofol (Propofol^®^Lipuro-vet, Virbac) until effect. The patient was intubated using an endotracheal tube of the size of 13. Maintenance of the general anesthesia was performed with isoflurane (Vetflurane^®^ 1000 mg/g, Virbac) to maintain adequate general anesthesia depth. Magnetic resonance images were acquired from a 1.5 Tesla MRI scanner (Magneton Sola, Siemens Healthineers, USA) in transverse, sagittal, and dorsal planes in T2w, FLAIR, SWI, and T1w pre- and post-contrast. Post-contrast images were obtained after administration of Gadovist ^®^ 1.0 mmol/L gadobutrol, Bayer ^®^. The slice thickness was 2.5 mm. MR images were initially performed from the T3 vertebra to the sacrum for investigations into the suspected T3-L3 myelopathy.

**Figure 1:**
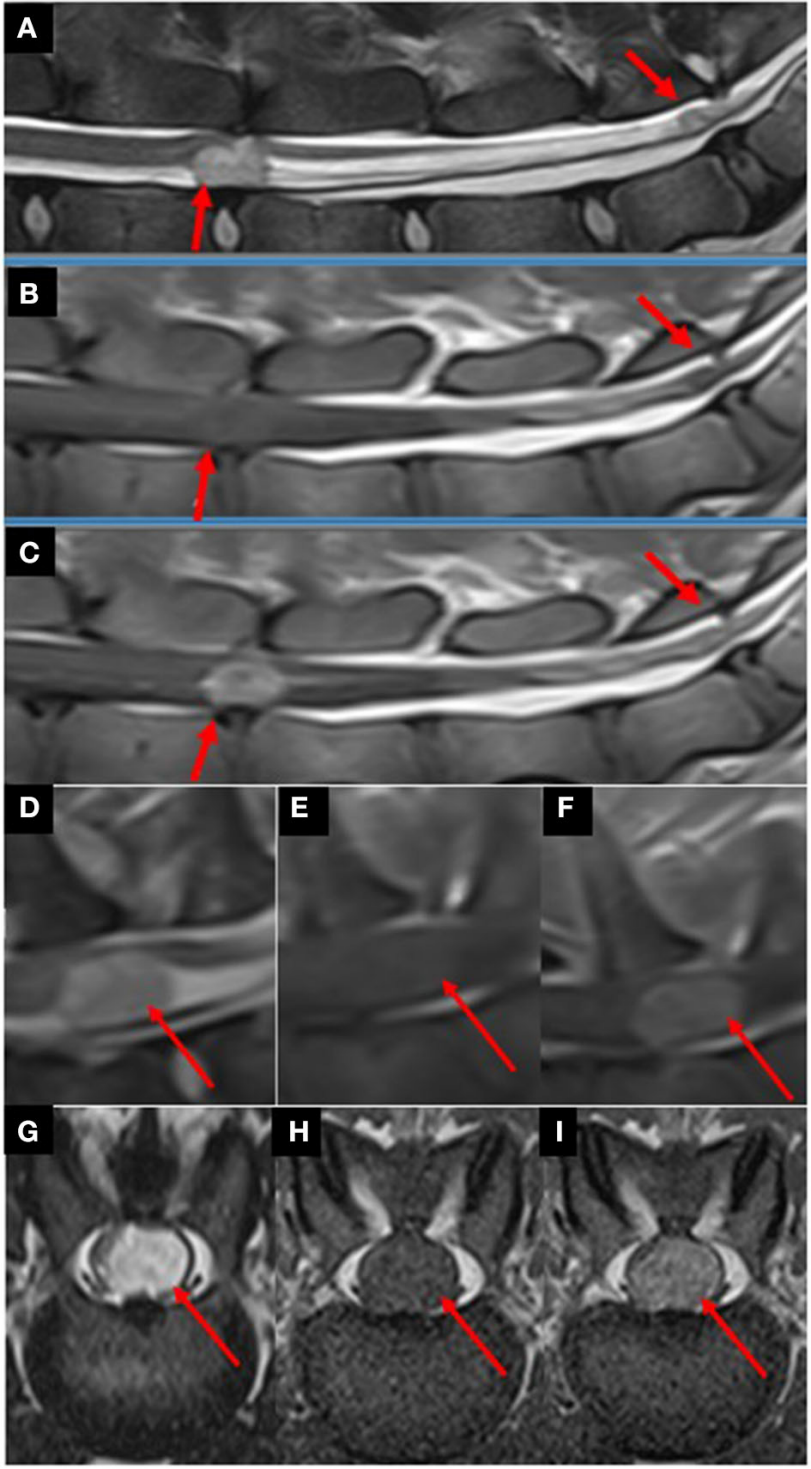
Parasagittal T2w **(A)**, T1w **(B)**, and T1w post-contrast **(C)** images from L4 to S1 vertebrae revealing multiple well-defined, round-to-ovoid intradural-extramedullary space-occupying lesions (red arrows) at the level of L4-L5 intervertebral disk space and at the level of the lumbosacral junction. Compared with spinal cord parenchyma, the lesions were heterogeneously markedly hyperintense in T2w, iso- to hypointense in T1w images. After contrast-medium administration, the lesions were markedly contrast-enhancing. The lesions were causing marked-to-severe spinal cord and cauda equina spinal nerve compression. Parasagittal T2w **(D)**, T1w **(E)**, and T1w post-contrast **(F)** images at the level of the C7 vertebra revealed a single well-defined, heterogenous, round-to-ovoid intradural-extramedullary space-occupying lesion (red arrows). Compared to spinal cord parenchyma, the lesion was heterogeneously markedly hyperintense in T2w, iso- to hypointense in T1w images. After contrast-medium administration, the lesion was markedly contrast-enhancing. The lesion is causing marked-to-severe spinal cord compression Transverse T2w **(G)**, T1w **(H)**, and T1w post-contrast **(I)** images at the level of L4-L5 intervertebral disk space revealing a left-sided intradural-extramedullary space-occupying lesion (red arrows) causing severe spinal cord compression. Compared to spinal cord parenchyma, the lesions were heterogeneously markedly hyperintense in T2w, iso- to hypointense in T1w images. After contrast-medium administration, the lesions were markedly contrast-enhancing.

Pre-contrast MRI from the T3 vertebra to the sacrum revealed two distinct, well-defined, sharply demarcated, non-invasive, heterogenous, and round-to-ovoid intradural-extramedullary space-occupying lesions at the level of L4-L5 and L7-S intervertebral disk spaces, respectively, measuring 1.2 cm × 0.8 cm and 0.3 cm diameter. Compared with spinal cord parenchyma, the lesions were heterogeneously markedly hyperintense in T2w, iso-to-hypointense in T1w images. The lesions were causing marked-to-severe spinal cord and cauda equina spinal nerve compression. The lesions were consistent with intradural-extramedullary neoplasia (Figure 1).

Owing to the suspicion of spinal CSF drop metastases, the pre-contrast MRI study was extended to include the remainder of the CNS. Pre-contrast MRI from the C1 vertebra to T3 vertebrae revealed a similar intradural-extramedullary space-occupying lesion at the level of the C7 vertebra, measuring 1.2 cm in diameter (Figure 1).

**Figure 2:**
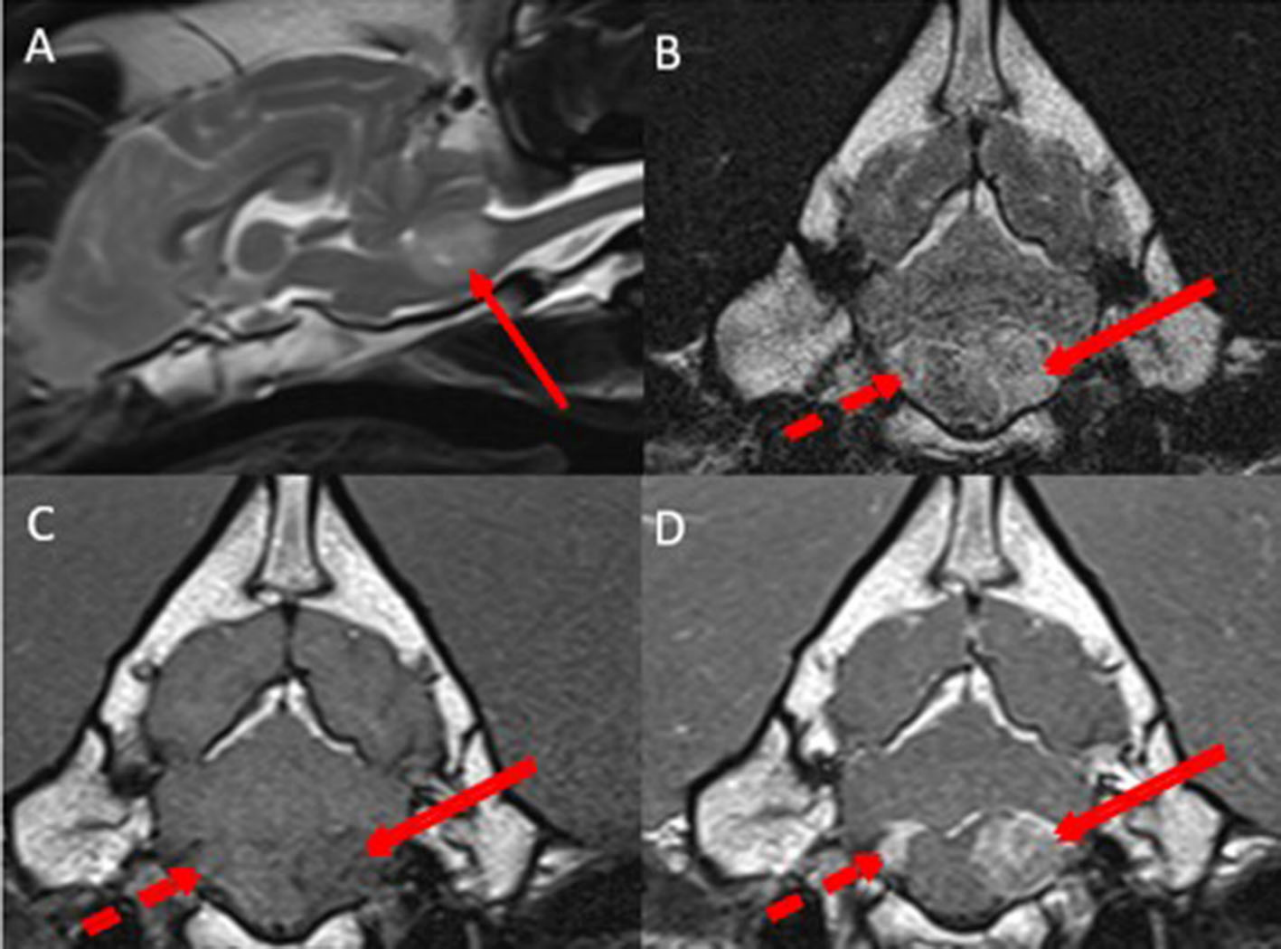
Midsagittal T2w **(A)**, transverse T2w **(B)**, transverse T1w **(C)**, and transverse T1w post-contrast **(D)** images at the level of the lateral apertures revealing bilateral asymmetrical well-defined, heterogenous, intraventricular space-occupying lesions affecting the left side of the fourth ventricle and the left lateral aperture (red arrows) and the right side of the fourth ventricle and right lateral aperture (dotted red arrow). Compared with gray matter, the lesions were heterogeneously hyperintense in T2w images, iso- to hypointense in T1w images. After contrast-medium administration, the lesions were markedly contrast-enhancing. The lesions were causing moderate cerebellar and medulla oblongata compression.

Pre-contrast MRI of the head revealed a round-to-ovoid intraventricular space-occupying lesion affecting the caudal left aspect of the fourth ventricle, extending into the left lateral aperture, measuring 1.8 × 1.6 cm in diameter. Compared to gray matter, the lesion was heterogeneously hyperintense in T2w, T2w FLAIR images, iso-to-hypointense in T1w images. No area of signal void could be observed in susceptibility-weighted images. The lesion was causing moderate cerebellar and medulla oblongata compression. A similar but smaller lesion could be observed in the caudal right aspect of the fourth ventricle, extending into the right lateral aperture (Figure 2).

Once the pre-contrast MRI from the head to the sacrum was acquired, contrast medium (Gadovist ^®^ 1.0 mmol/L gadobutrol, Bayer ^®^) was administered at 0.1 ml/kg IV, and post-contrast MRI from the head to the sacrum was performed. All the above lesions described were markedly homogenously contrast-enhancing (Figures 1, 2).

The main differential diagnosis was a neoplastic disease (such as choroid plexus carcinoma, oligodendroglioma, lymphoma, and ependymoma) and disseminated CSF drop metastases to the ventricular and spinal subarachnoid space.

Owing to the poor prognosis and the current poor quality of life, the owners were elected for euthanasia.

Consent for a post-mortem examination was obtained, and a post-mortem examination was performed. A post-mortem examination was performed on arrival, 2 days post-euthanasia, and tissues were fixed in 10% neutral buffered formalin. After allowing a week for complete fixation of the brain, both the brain and spinal cord were serially sectioned.

Macroscopic examination of the cardiovascular system, respiratory system, gastrointestinal tract, urogenital system, peripheral nervous system, endocrine system, and musculoskeletal and lymphatic systems did not reveal any abnormality.

**Figure 3:**
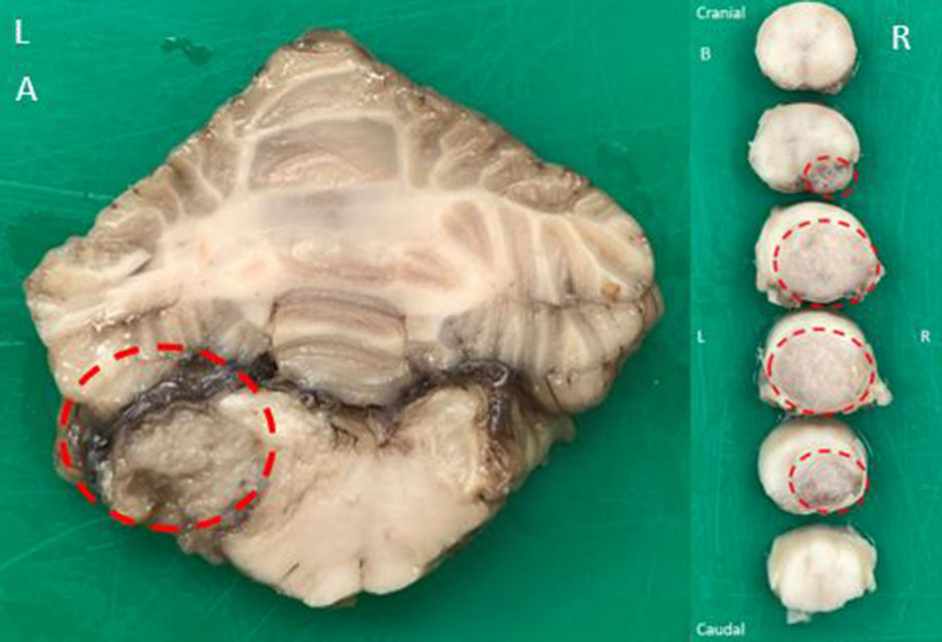
Macroscopic examination of a transverse fixed specimen of the patient's brain at the level of the lateral apertures **(A)** revealing a left-sided slightly irregular, soft, gray mass of maximum 1 cm in diameter (dotted red circle). The mass was compressing the adjacent medulla oblongata. Macroscopic examination of serial transverse fixed spinal cord segments at the level of C7 vertebra craniocaudally orientated **(B)** revealing a large, soft, gray, subdural mass present at the right ventral aspect of the spinal cord and expanding to displace almost the entire cord at its maximal limit (dotted red circles). There was severe spinal cord compression to approximately one-third of its original size.

Macroscopic examination of the brain revealed a left-sided slightly irregular, soft, gray mass of a maximum of 1 cm in diameter, located beneath the dura mater but appearing to be separated from the leptomeninges at the level of the left lateral aperture. This lesion compressed the medulla oblongata at the level of the left lateral aperture (Figure 3). A similar space-occupying lesion was identified, though smaller, at the level of the right lateral aperture.

Macroscopic examination of the spinal cord at the level of the C7 vertebra revealed a 2 cm in length soft gray subdural mass causing dorsolateral displacement of almost the entire spinal cord to the left side. There was severe spinal cord compression. The mass was displacing the leptomeninges rather than invading them (Figure 3). At the level of L4-L5 intervertebral disk space, a similar mass was compressing the spinal cord ventrally and on the left. At the level L7-S1 intervertebral disk space, another similar mass was displacing the cauda equina without invasion of the spinal nerves. All lesions shared a similar macroscopic appearance.

**Figure 4:**
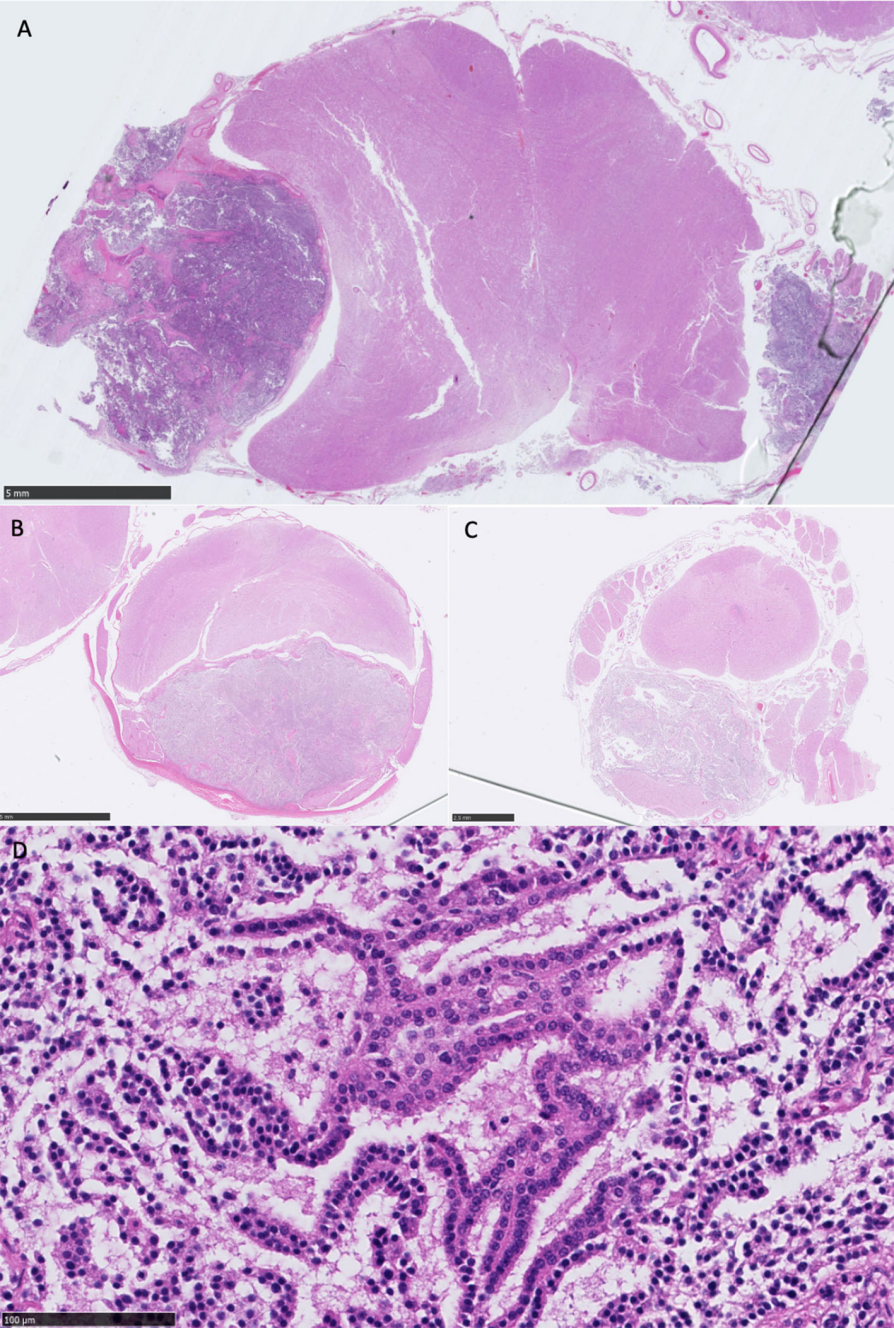
**(A)** Overview of medulla oblongata showing bilateral well-demarcated neoplastic masses, most prominent on the left, causing a midline deviation to the right, HE stain, scale bar 5 mm; **(B)** overview of the cervical intumescence and mass, enclosed by the dura mater, with severe dorsal compression of the entire spinal cord, HE stain, scale bar 2.5 mm; **(C)** overview of the lumbar spinal cord and mass, with dorsal compression of the spinal cord. A neoplastic aggregate is also visible at the center of the spinal cord, within the spinal canal, HE stain, scale bar 2.5 mm; **(D)** high-power view of the primary neoplasm showing single layer papillary formations (cell dehiscence present owing to autolysis), monomorphic nuclei, and no mitoses present in this field, representative of cell morphology observed, HE stain, scale bar 100 μm, equivalent to ×400.

Histological examination at the level of the medulla oblongata and cerebellum revealed a partially encapsulated, multifocally infiltrative, and moderately cellular neoplastic mass affecting the left of the medulla oblongata, originating from the choroid plexus of the fourth ventricle on that side. Although post-mortem artifact affected the preservation of tissue architecture (owing to areas of cell dehiscence), a papillary pattern was still clearly evident and individual cell morphology could still be assessed. The papillary pattern was almost exclusively single-layered supported by a fibrovascular stroma. Nuclei were small and hypochromatic. Toward the invasive regions, they were larger and coarsely stippled. There was otherwise little anisocytosis and anisokaryosis. Mitoses were rarely observed (1/2.37 mm^2^). The mass was compressing the adjacent medulla oblongata. There were multifocal hypereosinophilic (necrotic) Purkinje cells in the adjacent cerebellar folia. A much smaller similar mass was found, affecting the right side of the medulla oblongata (Figure 4).

The lesions in the spinal subarachnoid space were identical, and their histological examination demonstrated the same subdural neoplastic proliferation. The cells also formed more solid areas with a lack of supporting stroma, and there was multifocal necrosis. The mitoses were rare (1/2.37 mm^2^). In one section, a ventral spinal artery exhibited fibrinoid necrosis. There was widespread vacuolation and scattered spheroids, particularly in the region of ventral spinal tracts. The dura mater exhibited focal osseous metaplasia (Figure 4).

Morphologically, the neoplasia was most consistent with a primary fourth ventricle choroid plexus papilloma (CPP - grade I CPT), with disseminated CSF drop metastases to the ventricular (CPP- grade I CPT) and spinal (aCPP - grade II CPT) subarachnoid spaces.

## Discussion

Contrary to human medicine, the clinical cases of disseminated CSF drop metastases to the ventricular and spinal subarachnoid space secondary to CPP have not been yet reported in the veterinary literature (13, 14). To the author's knowledge, this is the first case report describing clinical, MRI, macroscopic, and histopathological features of primary intraventricular CPP with disseminated CSF drop metastases to the ventricular (CPP) and spinal (aCPP) subarachnoid spaces in a young adult dog.

According to the published literature in veterinary medicine, the median age of dogs diagnosed with CPP is 5 years (ranging from 3 to 14 years) (3, 4, 15, 17–23). Our patient is the youngest dog diagnosed with CPP. Depending on their intracranial location, reported clinical signs associated with CPP include epileptic seizures, vestibular syndrome, neck pain, and altered mentation and regurgitation (3, 4, 19–24). Our patient did not display any clinical signs consistent with intracranial disease. In the present report, our patients presenting chief complaints were ambulatory paraparesis, general proprioceptive ataxia, and spinal pain. Due to the patient's behavior, the thoracic limbs could not be reliably assessed. Assessment of a more compliant patient may have resulted in the identification of subtle proprioceptive deficits in the thoracic limbs and reduced spinal reflexes due to the C7 vertebra lesion.

The MRI features of CPPs have been documented in the veterinary literature (2–4, 10, 20). On MRI, CPPs appear as globular to papilliform intraventricular space-occupying lesions (2, 4, 10, 20). Compared to gray matter, CPPs are hyperintense in T2w images and hypo- to hyperintense in T1w images (2, 4, 10, 20). After contrast-medium administration, CPPs are strongly contrast-enhancing (2, 4, 10, 20). Ventriculomegaly is reported in 78% of intracranial CPPs (4). Periventricular hyperintensity observed on T2w FLAIR images is reported in all CPPs (4, 10). Ventriculomegaly and hydrocephalus in CPP result from the complex interaction of several factors, including the obstruction of the CSF flow and the increase in CSF production (25, 26). In this report, the intracranial CPPs corresponded to the literature description. However, no T2w FLAIR periventricular hyperintensity could be observed, and there was no sign of ventriculomegaly. This could be explained because the MRI was performed before these features could develop.

Spinal CPPs have been rarely documented in dogs and cats (15–17). Their MRI features are, therefore, only rarely described. Secondary spinal CPPs are reported as extramedullary-intradural space-occupying lesions (15–17). Compared to spinal cord parenchyma, spinal CPPs are hyperintense in T2w images and iso- to hypointense in T1w images (15–17). After contrast-medium administration, the lesions are markedly contrast-enhancing (15–17). These features were also present in our case. A research study on CSF drop metastases secondary to intracranial glioma identified that the CSF drop metastases may differ markedly from the primary mass on MRI (11). The authors reported that some nodules would show more homogeneous signals and less contrast enhancement than the primary lesion (11). This was particularly obvious in diffuse metastases to the leptomeninges compared to the primary intra-axial mass (11). In the present case, the primary CPP and disseminated CSF drop metastases to the ventricular and spinal subarachnoid space had the same MRI appearance.

According to a retrospective study on 56 CPT cases in dogs, the identification of disseminated CSF drop metastases to the ventricular and spinal subarachnoid space on MRI is reported as a reliable mean to clinically discriminate CPP from CPC (4). No CPP had subarachnoid metastasis, while 33% of CPC had subarachnoid metastases identified on MRI (4). However, the authors reported that one CPP presented a subarachnoid metastasis (4). The authors did not provide further information on the location of the metastasis and did not report any MRI evidence of metastasis in the CPP group. It could be suspected that the metastatic mass was only incidentally identified on post-mortem examination (4). To this date, there are only two case reports in dogs documenting histologically confirmed spinal subarachnoid CPP on MRI (15, 17). In the first case report on long-term outcomes after surgical resection of a spinal CPT, the authors' first MRI was focused on investigating a T3-L3 myelopathy (17). An intradural-extramedullary space-occupying lesion was identified, surgically debulked, and histologically analyzed (17). The histological features were consistent with a choroid plexus papilloma (17). Repeated MRI at 4 months post-surgery did not reveal any local recurrence (17). However, the wider field of view compared with the initial MRI revealed a space-occupying lesion affecting the right lateral aperture consistent with a CPT (17). Due to the absence of intracranial signs, no further investigations were performed (17). The patient died 25 months post-operation due to deterioration of intracranial signs and epileptic seizures (17). No post-mortem analysis was performed to confirm the suspected CPT (17). In the second report, the authors justified the absence of an MRI of the head as the patient did not present any intracranial signs and concluded that the spinal CPP identified on MRI and surgically debulked was of suspected ectopic origin (15). However, as emphasized by our case report and in another case report on disseminated choroid plexus carcinoma, the absence of intracranial signs cannot preclude the absence of intracranial lesions (27). Some authors have recommended that an MRI of the spinal cord is performed in cases of suspected CPT as 19% of CPCs have evidence of spinal cord metastases on post-mortem (4). On the contrary, the present case report and the two other case reports published in the veterinary literature should alert the clinician that a patient with CPP may not display intracranial clinical signs. A complete and extensive MRI study, including the entire CNS, should be performed in dogs presenting multifocal intradural-extramedullary space-occupying lesions.

Choroid plexus tumors are intraventricular neoplasia arising from the choroid plexus of the third and fourth ventricles (4). Macroscopically, CPPs are granular, rough textured, and circumscribed gray to reddish masses (8). On histological analysis, CPP recapitulates normal choroid plexus with papilliform fronds, with a dense connective core around a central vessel and covered by a single layer of cuboidal to columnar neoplastic epithelium (4, 5, 8). Mitotic figures are rare (4, 7, 8). The histological diagnosis of CPC relies on distinctive features such as frequent mitoses (> 5 per 10 high-power fields), nuclear atypia, increased cell density, focal loss of papillary formation with cell sheeting, necrosis, and increased layering of the epithelium (4, 5, 7, 8). In this report, the identified ventricular CPTs corresponded to the above CPP description. The spinal subarachnoid metastases shared similar histological features (such as rare mitotic count) with the primary neoplasia but also presented areas of loss of papillary pattern (solid areas) and necrosis. These two features could be sufficient to grade the CPT spinal subarachnoid metastatic lesions as aCPP, which would appear as a neoplastic progression from the primary fourth ventricular CPP (4, 5, 7).

A limitation of this case report is the absence of CSF analysis. Total protein concentration in the CSF has been described as an antemortem criterion for the differentiation between CPC and CPP in dogs (4). An increased total protein concentration above 80 mg/L in the CSF was consistent with a CPC diagnosis (4). In this case report, the absence of CSF analysis remains marginal due to the confirmed histopathological diagnosis of CPP and disseminated CSF drop metastases to the ventricular and spinal subarachnoid space. Another limitation of this case report is that post-mortem and histological examinations were performed 2 days after euthanasia and formalin fixation which may have allowed post-mortem and/or histological artifacts.

This case report provides the first description of clinical, MRI, and histological features of primary ventricular CPP and disseminated CSF drop metastases to the ventricular (CPP) and spinal (aCPP) subarachnoid space in a young adult dog. This case report should alert the clinician that the absence of intracranial signs cannot preclude the absence of intracranial lesions and highlights the importance to perform advanced imaging of the entire CNS to provide the owners with the full extent of the lesions, even for suspected primary benign neoplasia such as CPP.

## Data availability statement

The original contributions presented in the study are included in the article/supplementary material, further inquiries can be directed to the corresponding author.

## Ethics statement

Ethical review and approval was not required for the animal study because no ethical approval required for this case report. Consent was obtained for investigations, euthanasia and post-mortem examination from the owners directly. Written informed consent was obtained from the owners for the participation of their animals in this study. Written informed consent was obtained from the participant/patient(s) for the publication of this case report.

## Author contributions

GA wrote the manuscript. AM performed a post-mortem examination and histological analysis, provided a post-mortem report, and corrected the histological analysis content of the manuscript. AS reported the MR images and corrected the diagnostic imaging content of the manuscript. FS supervised, reviewed, revised, and corrected the manuscript prior to its submission. All authors contributed to the article and approved the submitted version.
